# Comparison of the Triage Micro Parasite Panel and Microscopy for the Detection of *Entamoeba histolytica/Entamoeba dispar*, *Giardia lamblia*, and *Cryptosporidium parvum* in Stool Samples Collected in Kenya

**DOI:** 10.1155/2012/564721

**Published:** 2012-07-10

**Authors:** Brett Swierczewski, Elizabeth Odundo, Janet Ndonye, Ronald Kirera, Cliff Odhiambo, Edwin Oaks

**Affiliations:** ^1^Kenya Medical Research Institute/Walter Reed Project, United States Army Medical Research Unit—Kenya, P.O. Box 1357, Kericho 20200, Kenya; ^2^Division of Bacterial and Rickettsial Diseases, Walter Reed Army Institute of Research, 503 Robert Grant Avenue, Silver Spring, MD 20910, USA

## Abstract

*Entamoeba histolytica*, * Giardia lamblia*, and *Cryptosporidium parvum* are three of the most important parasitic causes of acute diarrhea worldwide. Laboratory diagnosis of these parasites is usually done by ova and parasite examination (O&P examination) via microscopy. The sensitivity and specificity of O&P examination varies among laboratories and can be labor intensive and time consuming. The Triage Micro Parasite Panel (BioSite, San Diego, California) is an enzyme immunoassay kit that can detect *E. histolytica/E. dispar*, *G. lamblia*, and *C. parvum *simultaneously using fresh or frozen stool. The present study evaluated the Triage Micro Parasite Panel in detecting *E. histolytica/E. dispar*, *G. lamblia*, and *C. parvum *compared to O&P examination in 266 stool samples collected at medical facilities in Kenya. The sensitivity and specificity results for the Triage Micro Parasite Panel were: for *E. histolytica/E. dispar*: 100%, 100%, *G. lamblia*: 100%, 100% and *C. parvum*: 73%, 100%. There was no evidence of cross reactivity using the kit with other parasites identified in the stool specimens. These results indicate that the Triage Micro Parasite Panel is a highly sensitive kit that can be used for screening purposes in large scale studies or outbreak investigations or as a possible alternative to O&P examination.

## 1. Introduction

Diarrheal disease is a major cause of morbidity and mortality worldwide particularly in developing countries where poor sanitary and hygienic conditions exist [1–4]. The most common parasitic causes of acute diarrhea are the intestinal protozoa of which *Entamoeba histolytica*, *Giardia lamblia,* and *Cryptosporidium parvum* are considered the most important [5, 6]. Detection of trophozoites, cysts, or oocysts in fresh or preserved stool specimens using microscopy (ova and parasite examination (O&P)) is the most common method of diagnosis particularly in resource limited countries. Though microscopy is fairly inexpensive, it can be time consuming and labor intensive, and diagnosis usually depends on the microscopist's level of expertise and training [7–10]. Due to the technical variability among laboratory technologists, misidentification of these parasites, particularly *E. histolytica, *has led to unnecessary or delayed treatment [11, 12]. In addition, previous studies have shown that excretion of trophozoites, cysts, or oocysts in the feces can be intermittent and sporadic from day to day and therefore could lead to missed infections due to the low numbers of the diagnostic stages in the stool sample [13, 14]. For these reasons, several rapid enzyme immunoassay (EIA) kits have become available within the last 20 years for the detection of *E. histolytica*/*E. dispar*, *G. lamblia, *and *C. parvum *in stool specimens and have provided an alternative to O&P examination for detection of these parasites [9, 15–17].

The Triage Micro Parasite Panel (BioSite, San Diego, CA, USA) is an EIA kit which is able to simultaneously detect specific antigens of *E. histolytica*/*E. dispar*, *C. parvum,* and *G. lamblia *in stool. The assay can be read within 15 min using fresh or frozen unfixed stool samples. The assay has been compared to O&P examination for the detection of *E. histolytica*/*E. dispar*, *C. parvum,* and *G. lamblia *in several previous studies reporting sensitivities and specificities ranging from 91.5% to 100% [18–21]. To date, the Triage Micro Parasite Panel has not been evaluated in stool samples collected in East Africa. Here, we report the first evaluation of the Triage Micro Parasite Panel compared to O&P examination for the detection of *E. histolytica/E. dispar*, *C. parvum,* and *G. lamblia* in stool samples from patients evaluated at several hospitals in an outpatient setting in Kenya.

## 2. Materials and Methods

### 2.1. Specimens

A total of 266 (*n* = 266) stool specimens were used for this study that were collected as part of an ongoing acute diarrhea case-control study. Specimens were collected from both cases (*n* = 134) and controls (*n* = 132) from hospitals located in Nyanza, Rift Valley, and Central Provinces of Kenya. Cases were defined as those individuals having 3 or more episodes of loose, watery, or bloody stool in less than a 24 h period. Age-matched controls (reported for medical reasons other than acute diarrhea) were recruited from the same hospital in which the cases were seen and had no episodes of diarrhea within 2 weeks prior to enrollment. Both cases and controls were recruited from an outpatient setting only. After collection, aliquots of the stool specimen were placed in one vial containing 10% formalin and a clean vial without preservative. Both vials were stored immediately at 4°C at the collection sites. Specimens were packed in air tight cool boxes on ice packs for transport. Specimens were received at the microbiology laboratory located in Kericho, Kenya within 48 h after collection, immediately frozen at −20°C and analyzed within 48 h after receipt. All 266 samples were tested using both O&P examination and the Triage Micro Parasite Panel.

### 2.2. Triage Micro Parasite Panel

The Triage Micro Parasite Panel (Biosite Incorporated, San Diego, CA, USA) was used for all enzyme immunoassay testing for the detection of *E. histolytica*/*E. dispar*, *G. lamblia,* and *C. parvum* according to the manufacturer's instructions. Only frozen stool samples were used in the assay and were brought to room temperature before testing. All steps of the Triage Micro Parasite Panel were carried out at room temperature (15–25°C). The sample specimen was prepared by adding stool to the specimen tube (provided in the kit) which contained 4.5 mL of specimen diluents and vortexed for 10 s. A filter device provided in the kit was added to the specimen tube and centrifuged for 5 min at 1500 ×g. 0.5 mL of the filtered sample was then added to the center of the Triage device (test panel). Enzyme conjugate (140 *μ*L) was transferred directly to the center of the test panel and allowed to incubate for 3 min. The test panel was washed twice with seven drops of wash solution followed by the addition of four drops of substrate onto the center of the test panel. After a 5 min incubation period, the test panel was read. The production of color bars (purple) in the parasite specific test zones was interpreted as positive. Positive and negative controls supplied with the kit were prepared according to the manufacturer's instructions and used in every assay for quality control.

### 2.3. O&P Examination

Stool specimens stored in 10% formalin were concentrated using the Mini Parasep SF kit (DiaSys, Berkshire, England) according to the manufacturer's instructions. Briefly, stool was transferred to the Mini Parasep filter device, emulsified in 3.3 mL of 10% formalin, and incubated at room temperature for 24 h. After centrifugation of the filter device for 2 min at 500 ×g, the resulting supernatant located directly above the concentrated stool sediment was discarded. O&P examination consisted of analyzing two wet mounts prepared for each specimen (one stained with iodine) and analyzed by two laboratory technicians. For *C. parvum* identification, a modified acid-fast stain was used according to previously described methods and analyzed by two laboratory technicians [22]. For all O&P examination, control slides for *E. histolytica*/*E. dispar*, *G. lamblia,* and *C. parvum* were used and prepared along with the sample slides.

### 2.4. Discrepant Results

Those specimens with discrepant results were further analyzed using supervisory review. New specimens slides were prepared as described above. The laboratory supervisor and an additional laboratory technologist independently examined the slides of those samples with discrepant results. A true positive was defined as both the laboratory supervisor and the laboratory technologist positively identifying the parasite.

### 2.5. Statistics

McNemar's test was used for statistical analysis on case-control data. Sensitivity and specificity for O&P examination and the Triage Micro Parasite Panel were calculated according to Knapp and Miller [23].

### 2.6. Scientific Ethics

This study was performed under an existing protocol which was approved by the Walter Reed Army Institute of Research Institutional Review Board and the Kenya Medical Research Institute Ethical Review Committee. Informed consent was acquired from all participants, or if a minor, from their guardian or parents.

## 3. Results

### 3.1. Study Population

The median ages for cases and controls were 3 years, 4 months, and 3 years, respectively. 46% and 54% of the cases were male and female, respectively. 43% and 56% of the controls were male and female, respectively.

### 3.2. O&P Examination and Triage Micro Parasite Panel for *G. lamblia*

Positive specimens for *G. lamblia *were *n* = 20 (O&P examination) and *n* = 23 (Triage Micro Parasite Panel), respectively (Table 1). Supervisory review of the discrepant results indicated that there were three false negatives via diagnosis by O&P examination. The number of true positives after resolution of discrepant results was *n* = 23 and the number of true negatives was *n* = 243. *G. lamblia *was detected in 11 cases and 12 controls, respectively. The sensitivity and the specificity of the Triage Micro Parasite Panel were both 100% and 88% and 100%, respectively, for O&P examination (Table 2).

### 3.3. O&P Examination and Triage Micro Parasite Panel for *E. histolytica/E. dispar*

Positive specimens for *E. histolytica/E. dispar* were *n* = 12 (O&P examination) and *n* = 11 (Triage Micro Parasite Panel), respectively (Table 1). Supervisory review of the discrepant results indicated that there was one false positive via diagnosis by O&P examination. The number of true positives after resolution of discrepant results was *n* = 11, and the number of true negatives was *n* = 255. *E. histolytica/E. dispar *was detected significantly more often in the controls than in the cases (1 case versus 10 controls; *P* < 0.05). The sensitivity and the specificity of the Triage Micro Parasite Panel were both 100% and 100% and 99%, respectively, for the O&P examination (Table 2).

### 3.4. O&P Examination and Triage Micro Parasite Panel for *C. parvum*

Positive specimens for *C. parvum *were *n* = 8 (O&P examination) and *n* = 4 (Triage Micro Parasite Panel) initially. Supervisory review of the discrepant results indicated that there were 3 false-negatives via diagnosis by the Triage Micro Parasite Panel (Table 1). The number of true positives after resolution of discrepant results was *n* = 8, and the number of true-negatives was *n* = 258. *C. parvum* was detected in 4 cases and 4 controls, respectively. The sensitivity and the specificity of the Triage Micro Parasite Panel was 73% and 100%, respectively and 100% and 100% for O&P examination (Table 2).

### 3.5. Other Parasites Detected

Additional parasites detected by O&P examination included the following: *Ascaris lumbricoides *(*n* = 3), *Entamoeba coli *(*n* = 2), *Chilomastix mesnili *(*n* = 1), and *Hymenolepis nana *(*n* = 1) (Table 1).

## 4. Discussion

Laboratories in the developing world continue to rely on O&P examination as the gold standard for detection of intestinal parasites as it is relatively inexpensive and appropriate for resource-limited countries in the developing world. However, accurate diagnosis of intestinal parasites mostly is dependent on the level of expertise of the microscopist, and therefore the sensitivity and the specificity of O&P examination can vary from laboratory to laboratory [24]. As *E. histolytica*, *C. parvum,* and *G. lamblia *are considered the three most common parasitic causes of acute diarrhea worldwide, it is imperative that accurate diagnosis is correct as misdiagnosis can lead to missed treatment resulting in morbidity/mortality and the continued shedding of the parasites leading to increased transmission. For these reasons, EIA kits such as the Triage Micro Parasite Panel offer an acceptable alternative method to O&P examination for diagnosis of *E. histolytica/E. dispar*, *C. parvum,* and *G. lamblia*.

Here, we report the first evaluation of the Triage Micro Parasite Panel in stool samples collected in an outpatient setting in Kenya for the detection of *E. histolytica/E. dispar*, *C. parvum,* and *G. lamblia*. These parasites are endemic in Kenya although the prevalence of these parasites in any particular region in Kenya is likely to vary depending on factors such as the sanitation infrastructure, social culture, and availability of diagnostic and medical care [25–27]. Overall, the sensitivity for *E. histolytica/E. dispar*, *C. parvum,* and *G. lamblia* reported was 100%, 80%, and 100%, respectively, for the Triage Micro Parasite Panel while the specificity for each parasite was 100%. Sensitivity of routine O&P examination for the diagnosis of *E. histolytica/E. dispar*, *C. parvum,* and *G. lamblia* was 88%, 100%, and 100%, respectively, while specificity was 100%, 100% and 99%. The sensitivity and specificity results reported here for *G. lamblia *and *E. histolytica/E. dispar* are in accordance with results published in previous studies [18–21]. Garcia et al. and Sharp et al. reported sensitivity and specificity for the detection of *G. lamblia* and *E. histolytica/E. dispar* using the Triage Micro Parasite Panel ranging from 95.9% to 99.7% and 99.8% and 100%, respectively, in each study which is in accordance with the results reported here.

In this study, the sensitivity of the Triage Micro Parasite Panel for the detection of *C. parvum* was 73% as compared to O&P examination of 100%. A previous report showed that the Triage Micro Parasite Panel was dependent on parasite density for the detection of *E. histolytica/E. dispar* in order to accurately produce a positive result [28]. Similar to *E. histolytica/E. dispar*, a minimum amount of parasite density of *C. parvum* could be required for the Triage Micro Parasite Panel to be able to detect the specific antigen. These three samples could have contained low levels of *C. parvum* oocysts that the assay was unable to detect. The shedding of *C. parvum* oocysts in the stool can be sporadic, and the infective dose required can be quite low as well [29, 30]. Though the numbers of positive samples were not as high as in the study conducted by Garcia et al., they were comparable to other studies that have compared the sensitivity and specificity of O&P examination and the Triage Micro Parasite Panel [18, 19, 21].

The advantages of the Triage Micro Parasite Panel are that it is rapid (less than 15 min) and can be used with fresh or frozen unfixed stool, and results can be easily read and interpreted on the test device as compared to O&P examination. Furthermore, minimal training on the assay is required, and there is no real requirement for diagnostic parasitology expertise by the operator. Additionally, there was no cross-reactivity with the Triage Micro Parasite Panel with other intestinal parasites (*A. lumbricoides*, *E. coli*, *C. mesnili, *and *H. nana*) identified in stool samples via O&P examination as negative results were produced for these stool samples. A limitation to the assay as with O&P examination is that both are unable to differentiate between the pathogenic *E. histolytica* and the nonpathogenic *E. dispar* [31]. *E. histolytica/E. dispar* were found more often in the controls when compared to the cases. The controls could have been harboring *E. dispar* as opposed to the pathogenic *E. histolytica*. Additional tests can be used to differentiate these two species but were not performed in the current study [32–34]. More recently, the Tri-Combo EIA parasite screen (TechLab, Blacksburg, VA, USA) has been shown to be able to distinguish between antigens of *E. histolytica* and *E. dispar* in human fecal samples [35].

In conclusion, the Triage Micro Parasite Panel was highly sensitive and specific for the detection of *E. histolytica/E. dispar*, *C. parvum,* and *G. lamblia.* The kit could be used in Kenya as a screening tool in wide-scale prevalence studies or in suspected outbreak investigations due to its rapidity and simple procedure and no need for formal parasitology training. The Triage Micro Parasite Panel could be used in conjunction with O&P examination in Kenyan medical laboratories or possibly as an alternative method. Though multiplex real-time PCR assays have been developed for the simultaneous detection of *E. histolytica*/*E. dispar*, *G. lamblia,* and *C. parvum*, these assays could prove to be cost prohibitive for clinical laboratories in Kenya [36]. Ultimately, factors such as cost, medical infrastructure, and socioeconomic factors will ultimately determine the use of Triage Micro Parasite Panel in Kenya.

## Figures and Tables

**Table 1:** Comparison of the Triage Micro Parasite Panel and O&P examination results for diagnosis of *Entamoeba histolytica/E. dispar*, *Cryptosporidium parvum,* and *Giardia lamblia* in 266266 stool samples. Results comparing the Triage Micro Parasite Panel and O&P examination in 266 stool samples. The true positive results were determined after discrepant samples were analyzed. For *G. lamblia*, there were three false-negative results by O&P examination. For *E. histolytica/E. dispar*, there was one false-positive result by O&P examination. For *C. parvum*, there were three false-negative results by the Triage Micro Parasite Panel. Additional parasites detected by O&P examination were *Ascaris lumbricoides *(*n* = 3), *Entamoeba coli* (*n* = 2), *Chilomastix mesnili *(*n* = 1), and *Hymenolepis nana *(*n* = 1).

Parasite	Initial positive samples		True positives
	O&P examination	Triage panel	
*G. lamblia*	20	23	23
*E. histolytica/E. dispar*	12	11	11
*C. parvum*	8	5	8

**Table 2:** Sensitivity and specificity results for the Triage Micro Parasite Panel and O&P examination.

	O&P examination		Triage panel	
	Sensitivity (%)	Specificity (%)	Sensitivity (%)	Specificity (%)
*G. lamblia*	88	100	100	100
*E. histolytica/E. dispar*	100	99	100	100
*C. parvum*	100	100	73	100
